# Buffalo Medical College Commencement

**Published:** 1865-03

**Authors:** 


### BUFFALO MEDICAL COLLEGE COMMENCEMENT.

After invocation by the Rev. Dr. Clark, the following Report of the Council of the University was presented:

At a meeting of the Council of the University of Buffalo, held on the afternoon of the 21st ult., upon the recommendation of the Faculty and Curators of the Medical Department of the University, the degree of Doctor in Medicine was conferred upon the following gentlemen, viz:

Albert H. Smith, Fredonia, Chautauqua County, N. Y.
James D. Dillabough, Hagersville, Haldimand County, C. W.
Samuel W. West, Allen's Hill, Ontario County, N. Y.
Andrew Howell, Shannonville, Hastings County, C. W.
John L. Chapel, New Lyme, Ashtabula County, Ohio.
George P. Eddy, jr., Lewiston, Niagara County, N. Y.
Absalom Billingston, Clarence, Erie County, N. Y.
Samuel H. Bennett, Prattsburg, Steuben County, N. Y.
John K. Griffin, North East, Erie County, Pa.
Dennis D. Loop, North East, Erie County, Pa.
Gustavus J. Ackley, Conewango, Chautauqua County, N. Y.
Thomas J. Whitney, Sherman, Chautauqua County, N. Y.
Nathan L. Lusk, Walworth, Wayne County, N. Y.
Thomas J. McArthur, Eagle Village, Wyoming County, N. Y.
Albert H. Crawford, Corfu, Genesee County, N. Y.
Coleman N. Clark, New Lyme, Ashtabula County, Ohio.
Richard C. Rice, Howard Steuben County, N. Y.
Nathaniel Sweet, Alfred, Allegany County, N. Y.
William Byrns, Bronson, Branch County, Michigan.
William Foster, Buffalo, Erie County, N. Y.
Dighton L. Case, Howard, Steuben County, N. Y.
Benjamin F. Beardsley, Coventry, Chenango County, N. Y.
David S. Mills, Sparta, Elgin, C. W.
James H. Hewitt, Columbus, Warren County, Pa.
Ezra J. Graves, Herkimer, Herkimer County, N. Y.
George U. Gleason, Buffalo, Erie County, N. Y.
Lloyd Houghton, Java Village, Wyoming County, N. Y.
Julius Wenz, Buffalo, Erie County, N. Y.
Thomas R. Warren, Cowanesque Valley, Tioga County, Pa.
John H. Pickett, Buffalo, Erie County, N. Y.
Henry James, Belleville, Hastings County, C. W.
George M. Palmer, Pike, Wyoming County, N. Y.
Ebenezer H. Thurston, Utica, Oneida County, N. Y.
Melville C. Follett, Buffalo, N. Y.
Henry Lapp, Clarence, Erie County, N. Y.
Edwin R. Armstrong, Rochester, N. Y.
Hosea B. Goff, Almond. Alleghany County, N. Y.
Edwin M. Stillman, Almond, Alleghany County, N. Y.
Pliny P. Gordon, Bronson, Branch County, Michigan.
John J. Sawyer, Fairport, Monroe County, N. Y.
James M. Gross, Brighton, Northumberland County, C. W.
Orrin A. Tompkins, Sinclearville, Chautauqua County, N. Y.

George L. Lewis, Stockton, Chautauqua County, N. Y.
Tompkins L. Denike, Sinclearville, Chautauqua County, N.Y.
John B. Edwards, Pekin, Niagara County, N. Y.
Thomas G. Christy, New Castile, Lawrence County, Pa.
Reuben A. Drake, Oswayo, Potter County, Pa.
John C. Cheeseman, Holland, Erie County, N. V.
Henry J. Murphy, Caledonia, Haldimand County, C. W.
William P. Willis, Wenona, Marshall County, Illinois.

The following Theses are deemed worthy of honorable mention, viz:

A Thesis upon "The Blood and its Phenomena," by Gustavus J. Ackley, of Conewango, Cattaraugus County, N. Y.

A Thesis upon "Puerperal Fever," by Melville Cox Follett, of Buffalo, N. Y.

A Thesis upon "Mental and Moral Emotions as morbific and curative agents," by Edwin R. Armstrong, of Rochester, N. Y.

A Thesis upon "Army Surgeons," by William Byrns, of Bronson, Branch County, Michigan.

A Thesis upon "The Heart," by John Leander Chapel, of New Lyme, Ashtabula County, Ohio.

Of these several Theses, the Thesis upon "Army Surgeons" is recommended for publication, not on account of any relative superior merit, but because of its subject, "Army Surgeons," attracting more popular notice at this critical state of affairs.

Next followed the Address to the Graduates, by Prof. Moore, which was listened to with earnest attention by those to whom it was immediately directed, and by the audience generally. The following Valedictory Address, was delivered by George U. Gleason, of the graduating class:

*Fellow Classmates:*—The hour when we must say good bye, has come. The hour which is to sever the bonds which have connected us together as students, has now arrived. The hour in which we are to bid our teachers farewell is now with us, and when departed we feel the sad consciousness that this will be our last meeting in time, and that our next will be called by the Eternal Deity to the grand amphitheater of an unknown world.

During this hour the pleasant relations of teacher and student, of preceptor and pupil, will be forever sundered, and the morrow's sun will see us separated, never to be re-united this side of the grave.

A knowledge of this fact might lead us to prolong this meeting, but as this may not be, let us mark the spot upon the tablets of memory, that we may refer to it with pleasure in the future, for

> "There are moments in life which are never forgot,
> Which brighten and brighten as time steals away,
> They add a new charm to the happiest lot,
> And they smile on the gloom of the loveliest day."

Such a moment is the present. From this time we begin life anew; we assume new duties and new responsibilities; we have new anxieties and new fears. To-night we have received the first fruits of compensation of a long and arduous struggle and much patient toil. The right hand of fellowship from the physician and the degree of Doctor of Medicine.

We start from this point to build for ourselves a reputation; this is to be our stock in trade. Upon this the world places a vast estimate, and this is so nearly allied to, and dependent upon *character*, that we may pause a moment to consider how character may be best obtained.

In the first place, no one can expect to practice successfully a profession which is based in science, nourished by science, and is alone to be perfected by science, without first having its deep underlying, scientific principles firmly established in his mind.

To this end we have had impressed upon our minds the necessity of a thorough knowledge of the physical nature of man, his anatomy and his physiology; the knowledge of plants, their habits, their growth, and their influence on man; the knowledge of the earth and minerals; and chemistry, both organic and inorganic; and the familiar use of the scalpel, the test tube, and the microscope. Neither can it be expected that a science which requires of its votaries the universal practice of honesty, bravery, charity, patience, temperance, purity and truth, can be practiced without first having a high, pure and umblemished character.

For this grand and ultimate foundation we must depend only upon ourselves. We are the sole architects of our character, and be it good or bad, it becomes the man.

Shall we call character and reputation synonymous terms? No, far otherwise. The one is what a man really *is*, while the other is what the *world thinks him to be.* Cowards sometimes assume the part of brave men, and villains will often appear moral. The true and the good have been persecuted as imposters, and the great and learned for advancing hitherto unrecognized truth, have been imprisoned as mad-men. This, however, is but temporary; we generally see a truthful correspondence presented between reputation and character. There is an undefined law by which we understand the true worth and value of those we are brought in contact with. The outward man being a reflection of the inward, the head being balanced by the heart, and every motion being prompted by an inward monitor, we soon learn to interpret faithfully the hidden springs of action by the outward manner. To gain this inward furnishment then, should be our primary object, sought first, last, continually, and a commensurate reputation will follow as sure as night follows day. The acquisition of a reputation alone should not solely nor chiefly engross our thoughts and command our energies. Be ours a nobler ambition, to cultivate those virtues upon which alone any just and worthy reputation can be established.

Thus having in actual possession those qualities of mind and heart which entitle to fame and success, we may ever preserve a manly self-respect, and cherish a proud consciousness of deserving well of fortune. And herewith there is always associated a calm and sustaining faith in coming success and reward, which no present neglect or failure can beat down or destroy. Hope ever remains to cheer and support the man of real merit, the man who makes wisdom and goodness the basis of his claims upon the esteem and patronage of his fellow men.

But, on the other hand, he who bends his energies primarily to gain reputation and achieve success, is tempted to neglect the legitimate means by which they are secured, and to resort to the pretensions of quackery and the arts of the empiric. He is elevated and depressed by the slightest turns of fortune; and when once the weakness of his arts and the hollowness of his pretensions are discovered, his failure is complete, and his fall final.

*Officers and Teachers:*—As we turn to take leave of you, it becomes our duty and our pleasure to acknowledge that you have faithfully labored, during the period in which we have enjoyed your instruction, to impress upon our minds the philosophy of honorable success, and to furnish us with the requisite knowledge, experience and skill, to commence and prosecute our profession with profit to ourselves, honor to our *Alma Mater*, and benefit to society. Whether we do well or ill in life, we confess that naught but praise can be awarded you. You have done your whole duty to us; and we shall always remember you with respect, gratitude and love.

And now, *fellow students*, hastens the moment when the word must be spoken which is at once the expression of mutual regard and wishes for each other's welfare, and the signal of final separation, and entrance upon our professional career. Let the ideal aim of our profession—the alleviation of human woes—awaken our noblest enthusiasm, and constitute the grand motive of our highest exertions. So that having acted well our respective part here on earth, we may meet at last in the future world together to share the rewards of good samaritans and faithful servants—FAREWELL.

